# The Pathogenesis of Coronavirus Disease 2019 (COVID-19): Evaluation and Prevention

**DOI:** 10.1155/2020/1357983

**Published:** 2020-07-10

**Authors:** Hayat Ouassou, Loubna Kharchoufa, Mohamed Bouhrim, Nour Elhouda Daoudi, Hamada Imtara, Noureddine Bencheikh, Amine ELbouzidi, Mohamed Bnouham

**Affiliations:** ^1^Laboratory of Bioresources, Biotechnology, Ethnopharmacology and Health, Faculty of Sciences, Mohammed First University, Oujda, Morocco; ^2^Faculty of Arts and Sciences, Arab American University Palestine, P. O. Box 240, Jenin, State of Palestine; ^3^Faculty of sciences, Mohamed First University, Morocco

## Abstract

Coronavirus Disease 2019 (COVID-19) has become a major health problem causing severe acute respiratory illness in humans. It has spread rapidly around the globe since its first identification in Wuhan, China, in December 2019. The causative virus is called severe acute respiratory syndrome coronavirus 2 (SARS-CoV-2), and the World Health Organization (WHO) named the new epidemic disease Coronavirus Disease (COVID-19). The incidence of COVID-19 continues to increase with more than three million confirmed cases and over 244,000 deaths worldwide. There is currently no specific treatment or vaccine against COVID-19. Therefore, in the absence of pharmaceutical interventions, the implementation of precautions and hygienic measures will be essential to control and to minimize human transmission of the virus. In this review, we highlight the epidemiology, transmission, symptoms, and treatment of this disease, as well as future strategies to manage the spread of this fatal coronavirus.

## 1. Introduction

Coronaviruses belong to the Coronaviridae family in the Nidovirales order. Corona represents crown-like spikes on the outer surface of the virus; thus, it was named coronavirus. Coronaviruses are minute in size (65-125 nm in diameter) and contain a single-stranded RNA as nucleic material, with a size ranging from 26 to 32 kilobases (kb) in length. The subgroups of the coronavirus family are alpha (*α*), beta (*β*), gamma (*γ*), and delta (*δ*) [1]. Several coronaviruses can infect humans, like the globally endemic human coronaviruses HCoV-229E, HCoV-NL63, HCoV-HKU1, and HCoV-OC43 that tend to cause mild respiratory disease, and the zoonotic Middle East respiratory syndrome coronavirus (MERS-CoV) and severe acute respiratory syndrome coronavirus (SARS-CoV) that have a higher case fatality rate [2]. In late December 2019, a cluster of patients was admitted to hospitals with an initial diagnosis of pneumonia of an unknown etiology. These patients were epidemiologically linked to a seafood and wet animal wholesale market in Wuhan, Hubei Province, China [3, 4]. The pathogen has been identified as a novel coronavirus. Initially tentatively named 2019 novel coronavirus (2019-nCoV), the virus has now been named SARS-CoV-2 by the International Committee of Taxonomy of Viruses (ICTV) [5]. This virus can cause the disease named coronavirus disease 2019 (COVID-19) [5]. The SARS-CoV-2 belongs to the same coronavirus group (Betacoronavirus) as SARS and MERS viruses that caused two of the more severe epidemics in recent years. As with SARS and MERS, this new coronavirus, 2019-nCoV, is believed to be of zoonotic origin, but may also be transmitted through the respiratory tract, by direct contact, and possibly via patients excreta which may contain the living virus [6]. Since the emergence of the 2019 novel coronavirus (2019-nCoV) infection in Wuhan, China, it has rapidly spread across China and many other countries [7]. The outbreak of COVID-19 has affected more than three million patients in 187 countries, areas, or territories with a mortality rate of 4.20% and has become a major global health concern [8]. Based on the evidence of a rapidly increasing incidence of infections and the possibility of transmission by asymptomatic carriers [9, 10], SARS-CoV-2 can be transmitted effectively among humans and exhibits high potential for a pandemic [11–13]. To date, the disease has spread worldwide and become a serious infectious disease affecting human health worldwide [14]. In the absence of specific therapeutic drugs or vaccines for 2019 novel coronavirus disease (COVID-19), it is essential to detect the diseases at an early stage and immediately isolate the infected person from the healthy population. In this paper, we attempt to review and document the current data related to Corona Virus Disease 2019 (COVID-19) including etiology, epidemiology, clinical characteristics, and measures of treatment of COVID-19, with a special focus on infection control and prevention.

## 2. Epidemiology of COVID-19

**Figure 1:**
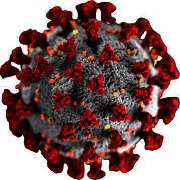
A graphical representation of the ultrastructural morphology of coronavirus (SARS-CoV-2). Source: Centers for Disease Control and Prevention—Public Health Image Library. Credit: Alissa Eckert, MS, Dan Higgins, MAM (Public Domain).

In December 2019, Wuhan City, Province of China, became the center of an outbreak of novel contagious coronavirus disease (COVID-19) of unknown etiology [11, 15]. Efforts are underway to continue to better understand more about transmissibility, severity, and other features associated with COVID-19 [16]. It appears that an infected animal may have first transmitted the virus to humans at a seafood market [17, 18]. Soon, a secondary source of infection was found to be human-to-human transmission of the COVID-19 virus [19]. It became clear that the COVID-19 infection occurs among close contacts and exposure to the virus [17]. Recent studies showed that people aged ≥ 60 years and the population with poor immune function such as diabetes, cardiovascular disease, chronic respiratory disease, cancer, renal, and hepatic dysfunction are at higher risk for severe COVID-19 than children who might be less likely to become infected or, if so, may show milder symptoms or even asymptomatic infection [20]. Coronavirus disease 2019 (COVID-19) is spreading rapidly across China and is being exported to a growing number of countries, some of which have seen onward transmission. According to the World Health Organization (WHO), COVID-19 continues to emerge and represents a serious problem to public health. On 2 May of March 2020, more than three million confirmed cases of COVID-19 reported by the World Health Organization. Of these, more than 240 000 have been fatal. About 83,959 cases were confirmed in China, and 4637 deaths were confirmed (Figure 1) [8]. The growing global tally includes spikes in Korea, Iran, Italy, Spain, France, and Germany. The virus is also continuing to spread to African countries including Algeria, South Africa, Senegal, Burkina Faso, Cameroon, Nigeria, and Côte d'Ivoire. In addition to the confirmed case, Moroccan's health ministry says that Morocco has more than 4500 confirmed cases of the coronavirus.

## 3. Origin and Transmission of COVID-19

**Figure 2:**
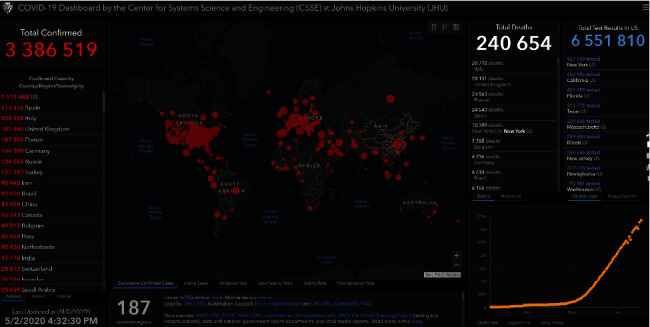
World map represents the geographical distribution of COVID-19 outbreaks. Data accurate as of 21 March 2020 [8].

The SARS-CoV-2 was found to be a positive-stranded RNA virus belonging to the genus Betacoronavirus with a crown due to the presence of spike glycoproteins on the envelope (Figure 2) [7]. Other than SARS-CoV-2, there are six types as humans coronaviruses have been identified, namely, HCoV-229E, HCoV-OC43, SARS-CoV, HCoV-NL63, HCoV-HKU1, and MERS-CoV [21]. Phylogenetic analysis revealed that the SARS-CoV-2 is closely related, with 88-89% similarity, to two bat-derived severe acute respiratory syndrome- (SARS-) like coronaviruses, bat-SL-CoVZC45 (accession no. MG772933.1), and bat-SL-CoVZXC21 (accession no. MG772934.1), but it is more distant from SARS-CoV, with about 79% similarity, and MERS-CoV, with about 50% similarity [22–24]. The SARS-CoV-2 has an envelope; its particles are round or elliptic and often polymorphic form, and a diameter of 60 nm to 140 nm [25]. Additional studies based on the genetic sequence identity and the phylogenetic reports confirmed that COVID-19 is different from SARS-CoV, and it can thus be considered as a new betacoronavirus that infects humans [26].

The source of the 2019-nCoV is still unknown. However, the growing outbreak has been linked to the Huanan South China Seafood Market [27]. Scientists are trying to find the animal host of this novel coronavirus in hopes of eradicating the spread, but so far, no one is certain. Most sources agree that the possible host of the 2019-nCoV is bats, pangolins, or seafood [3, 4, 25]. The task at hand is to find the intermediate host that is responsible for transmitting the coronavirus to humans. It is important to determine the source of the virus, to help the discovery of the zoonotic transmission patterns [25]. SARS-CoV-2 presents a high transmissibility and pathogenicity [28]. It could be transmitted from human to human by droplets and contact [28]. Several reports have suggested that symptomatic people are the most frequent source of COVID-19 spread. It primarily spreads between people through respiratory droplets by coughing or sneezing from an infected individual [26]. Moreover, there are suggestions that individuals who remain asymptomatic could transmit the virus. Further, studies are needed to clarify and understand the mechanisms of transmission, the incubation period, and the duration of infectivity of this virus.

## 4. Clinical Characteristics of COVID-19

In patients with Coronavirus disease 2019 (COVID-19), the most common clinical symptoms are fever and cough, shortness of breath, and other breathing difficulties in addition to other nonspecific symptoms, including headache, dyspnea, fatigue, and muscle pain [29, 30]. Moreover, some patients also report digestive symptoms such as diarrhea and vomiting [11, 30]. COVID-19 was similar to SARS and MERS in some clinical manifestations [29]. Fever occurred in 98-100% of patients with SARS or MERS, compared to 81.3% of patients with COVID-19 [29, 31, 32]. 18.7% of patients had no fever at admission, suggesting that the absence of fever could not rule out the possibility of COVID-19 [29]. Although patients initially have fever with or without respiratory symptoms, various degrees of lung abnormalities develop later in all patients, and these can be seen on chest CT (CT) [11, 33]. Although diarrhea is present in approximately 20-25% of patients infected with MERS-Cov or SARS-Cov, intestinal symptoms have rarely been reported in patients with COVID-19 [34]. Patients receive chest CT scans that provide reliable data on the dynamic X-ray pattern. Typical mild COVID-19 pneumonia begins primarily with small, subpleural, unilateral, or bilateral frosted glass opacities in the lower lobes, which then develop into a crazy-paving pattern and subsequent consolidation. After more than two weeks, the lesions are gradually absorbed with residual frosted glass opacities and subpleural parenchymal bands. In these patients who have recovered from COVID-19 pneumonia [35]. At admission, the majority of patients had lymphopenia and platelet abnormalities, neutrophils, aspartate aminotransferase (AST), aspartate aminotransferase (AST), lactate dehydrogenase (LDH), and inflammatory biomarkers. According to the results of the CT or X-ray, the patients had bilateral pneumonia and pleural effusion that occurred in 10.3% of the patients. Compared to patients in general, refractory patients had a higher level of neutrophils, AST, LDH, and reactive protein C and a lower level of platelets and albumin. In addition, refractory patients had a higher incidence of bilateral pneumonia and pleural effusion [36]. In general, hospitalized patients are classified in two categories, the general COVID-19 which has been defined according to the following criteria: obvious relief of respiratory symptoms (for example, cough, chest distress, and shortness of breath) after treatment, maintaining normal body temperature for more than three days without the use of corticosteroids or antipyretics, improving radiological abnormalities in the chest scanner or X-rays after treatment, a hospital stay of less than 10 days. Otherwise, it was classified as COVID-19 refractory. In the admission severity assessment, a serious illness was defined if it met at least one of the following: respiratory rate 30/min, pulse oximeter oxygen saturation (Spo2) 93% at rest, and partial arterial oxygen pressure (PaO2) at the inspired oxygen fraction (Fio2) 300 mmHg [29].

## 5. Treatment of COVID-2019

After the diagnosis of SARS-Cov2 infection was made, the prevention and quarantine are considered as the most way to stop the fast spreading of the virus, because there is no effective vaccine, drugs, or antiviral to prevent and treat this disease despite the great efforts made by the scientists and researchers around the world to develop vaccines and treatments of coronavirus. Furthermore, several strategies were carried out to help patients with COVID-2019 as oxygen therapy (major treatment intervention), antivirals (Lopinavir, Ritonavir, Ribavirin, Favipiravir (T-705), remdesivir, oseltamivir, Chloroquine, and Interferon) [25, 37, 38]. Most importantly, unselective or inappropriate administration of antibiotics should be avoided. Moreover, corticosteroids treatment should not be given for the treatment of SARS-Cov2 [39]. Convalescent plasma can be used to help people recover from viral infection without the occurrence of severe adverse events [40].

Among the difficulties that avoid finding the treatment for COVID-2019 is that the spike protein of the virus interacts with the host cell receptor including GRP78 (Glucose Regulating Protein 78). Consequently, the inhibition of this interaction would probably decrease the rate of the infection [41]. Lopinavir (protease inhibitor used to treat HIV) or Lopinavir/Ritonavir has shown *in vitro* anti-coronavirus activity [42]. In addition, the utilization of Lopinavir/Ritonavir showed a reduction of viral loads and it was found that it is able to improve virus symptoms during the treatment period [43]. Other reported antiviral treatments form human pathogenic CoVs include neuraminidase inhibitors like oral oseltamivir has been used in China hospitals for COVID-2019 cases [44]. No study has demonstrated the effectiveness of oseltamivir in the treatment of SARS-CoV-2 [42]. In Wuhan, on 6 February 2020, a clinical trial was initiated of remdesivir (Newly discovered antiviral drug) on SARS-CoV-2. This compound showed an inhibition of the replication of SARS-CoV and MERS-CoV in tissue cultures and efficacy in animal models [45]. However, given the related issues of security, safety, and efficacy, it is necessary to take some time to develop the vaccine and the antiviral drugs [46].

For a thousand years, Traditional Chinese medicine has gained an important experience in the infection healing. Currently, this kind of medicine has provided significant therapies for many current diseases as A H1N1 Influenza, A H7N9 Influenza, Ebola virus, and SARS-CoV [46–48]. Consequently, it can be also developed and applied in the treatment of COVID-2019. In fact, the decoction combination of Ma Xing Gan Shi (Combination includes *Ephedrae herba*, *Armeniacae semen amarum*, *Glycyrrhizae radix and rhizoma*, and *Gypsum fibrosum*) with Da Yuan Yin that includes *Arecae semen*, *Magnoliae officinalis* cortex, *Tsaoko fructus*, *Anemarrhenae rhizoma*, *Dioscoreae rhizoma*, *Scutellariae radix*, *Glycyrrhizae radix*, and *rhizoma* had showed in 2003 an important and a significant impact on SARS. The State Administration of Traditional Chinese Medicine advised on 6 February 2020, the utilization of Qing Fei Pai Du decoction that includes *Ephedrae herba*, *Gypsum fibrosum*, *Pinelliae rhizoma*, *Aurantii fructus immaturus*, and *Zingiberis rhizoma recen.* This decoction has been shown to be 90% effective in the treatment of SARS-CoV-2 [46, 49]. Besides, other Chinese herb combinations have been used to treat SARS-CoV infection like

- Yin Qiao San composed with Fructus Forsythiae, Flos Lonicerae, Radix Platycodonis, Herba Menthae, Herba Lophatheri, Radix Glycyrrhizae, Herba Schizonepetae, Fermented soybean, Fructus arctii, and Rhizoma Phragmitis
- Yu Ping Feng San includes Astragali radix, Astragalus membranaceus, Atractylodes macrocephala, and Saposhnikoviae Radix
- Shuang Huang Lian includes Lonicera japonica, Scutellaria baicalensis, and Forsythia suspensa
- Lian Hua Qing Wen Capsule includes Forsythia suspensa, Ephedra sinica, Lonicera japonica, Isatis indigotica, Mentha haplocalyx, Dryopteris crassirhizoma, Rhodiola rosea, Gypsum Fibrosum, Pogostemon cablin, Rheum palmatum, Houttuynia cordata, Glycyrrhizae, uralensis, and Armeniaca sibirica. This treatment can be used to control fever, cough, and tired related with COVID-19 [49]

Actually, there is no specific treatment or vaccine of COVID-2019; all of the drug options come from experience treating influenza, HIV, SARS, or MERS. At present, current efforts are focused on developing vaccines or specific antiviral drugs for COVID-19.

## 6. Infection Control and Prevention of COVID-19

According to what was published by the World Health Organization and a number of international health institutes, there are many restrictions that must be followed, either on a personal level or on the environmental level, including early recognition by the patients; carrying out additional precautions for persons suspected of infection, as well as for people who had contact with patients before their patients were revealed; applying standard precautions for all patients and imposing administrative measures from various authorities, such as the environment and health authorities [50, 51]. In the current situation and to limit the spread of the COVID-19 virus, all countries should publish an awareness declaration of the symptoms of infection in all cities, especially in remote areas. Also, publish the easiest and fastest way for the methods that every patient should follow in the event of a patient. In addition, encourage HCWs to have a high level of clinical suspicion [52, 53]. The WHO confirmed that the rational, correct, and consistent use of personal protective equipment (PPE) also helps reduce the spread of pathogens. PPE effectiveness depends strongly on adequate and regular supplies, adequate staff training, appropriate hand hygiene, and appropriate human behaviour [51, 54, 55].

At the level of additional precautions for patients, patients should be isolated in private quarantine rooms; everyone who contact with the patients, whether family, friends, or visitors, should be placed in a quarantine and a distance for contacting between them should be established [56]; the patients should cover their mouth and nose during sneezing by using masks or tissue as well as the persons COVID-19 suspected should place medical masks in public places and closed rooms and after every sneeze; the patient must wash their hands well (with an alcohol-based hand rub or with soap and water), as a result of coming into contact with respiratory secretions; a proper and careful approach should be taken to eliminate all waste from patient uses [55], reducing as much as possible the exchange of equipment between patients and sterilizing them well when transporting them from one patient to another and after patient care, appropriate doffing and disposal of all PPE and hand hygiene should be carried out [54]. At the level of additional precautions for health care workers (HCWs), a specialized team must be identified to deal with the patients to limit the spread of infection through protection methods, including the use of a medical mask, use of gloves, wearing of eye protection or facial protection, and wearing a clean, nonsterile, and long-sleeved gown; health care workers are prohibited from touching their eyes and nose with gloves or uncovered hands and limit the number of HCWs, family members, and visitors who are in contact with suspected or confirmed COVID-19 patients [54, 55]. At the level of additional precautions for the environment surrounding patients, the surfaces and places that patients come in contact with should be sterilized regularly; ensure adequate ventilation in the health care facility; separation of at least one meter should be maintained between all patients and manage laundry, food service utensils, and medical waste in accordance with safe routine procedures [57, 58]. The last section is on administrative policies and regulations that include educating caregivers on how to dealing patients, developing policies and plans through which early recognition of acute respiratory infection potentially caused by COVID-19 virus, preventing overcrowding in public places as much as possible, ensuring that the necessary equipment for health care is provided in sufficient quantities and permanently, providing protection to quarantine areas by the authorities to reduce patients' contact with healthy people, and imposing sanctions on those who violate the provisions that have been put in place by the authorities to limit the spread of the COVID-19 virus [56].

## 7. Conclusion

In conclusion, COVID-19 has become a high risk to the general population and healthcare workers worldwide. However, scientific research is growing to develop a coronavirus vaccine and therapeutics for controlling the deadly COVID-19. Hence, health education on knowledge for disease prevention and control is also important to control and reduce the coronavirus infection rate. Further research should be directed toward the study of SARS-CoV-2 on animal models for analyzing replication, transmission, and pathogenesis in humans.

## Abbreviations

WHO: World Health Organization
Covid-19: Coronavirus disease 2019
SASRS-Cov2: Severe acute respiratory syndrome coronavirus 2
SARS-Coc: Severe acute respiratory syndrome coronavirus
MERS-Cov: Middle East respiratory syndrome coronavirus
HCov: Human coronavirus
ICTV: International Committee of Taxonomy of Viruses
CT: Computed tomography
HIV: Human immunodeficiency virus
PPE: Personal protective equipment
HCW: Health care workers.
